# Double-edged roles of ferroptosis in endometriosis and endometriosis-related infertility

**DOI:** 10.1038/s41420-023-01606-8

**Published:** 2023-08-22

**Authors:** Yangshuo Li, Yalun He, Wen Cheng, Zhihao Zhou, Zhexin Ni, Chaoqin Yu

**Affiliations:** 1 Department of Gynecology of Traditional Chinese Medicine, the First Affiliated Hospital of Naval Medical University, 200433 Shanghai, China; 2 Department of Pharmaceutical Sciences, Beijing Institute of Radiation Medicine, 100850 Beijing, China

**Keywords:** Cell death, Infertility

## Abstract

Endometriosis is strongly associated with infertility. Several mechanisms have been reported in an attempt to elucidate the pathophysiological effects that lead to reduced fertility in women with endometriosis. However, the mechanisms by which endometriosis affects fertility have not been fully elucidated. Ferroptosis is a novel form of nonapoptotic cell death that is characterized by iron-dependent lipid peroxidation membrane damage. In past reports, elevated iron levels in ectopic lesions, peritoneal fluid and follicular fluid have been reported in patients with endometriosis. The high-iron environment is closely associated with ferroptosis, which appears to exhibit a double-edged effect on endometriosis. Ferroptosis can cause damage to ovarian granulosa cells, oocytes, and embryos, leading to endometriosis-related infertility. This article summarizes the main pathways and regulatory mechanisms of ferroptosis and explores the possible mechanisms of the formation of an iron-overloaded environment in endometriotic ectopic lesions, peritoneal fluid and follicular fluid. Finally, we reviewed recent studies on the main and potential mechanisms of ferroptosis in endometriosis and endometriosis-related infertility.

## Facts

1. There is a high iron level in both peritoneal and follicular fluid in patients with endometriosis.
2. A high-iron environment may be key to triggering ferroptosis.
3. Ferroptosis may have a double-edged effect on the development of endometriosis.
4. Ferroptosis impairs the function of oocytes and granulosa cells in patients with endometriosis.

## Open questions

1. How does endometriosis tolerate the high levels of iron in the peritoneal fluid?
2. Could ferroptosis inducers be the next potential treatment for endometriosis?
3. How do iron overload and ferroptosis affect endometriosis-related infertility?
4. How can ferroptosis be balanced to treat endometriosis and endometriosis-related infertility?

## Introduction

Endometriosis refers to an oestrogen-dependent inflammatory disease characterized by the seeding and growth of endometrial tissue outside the uterine cavity [1]. These endometrial tissues can be seeded on the peritoneal cavity, ovaries, and fallopian tubes, as well as distant tissues and organs [2]. The simultaneous detection of endometrial stromal and glandular components in histological biopsies is necessary to ascertain endometriosis [3]. The common clinical symptoms of endometriosis include chronic pelvic pain and infertility, which severely affect the physical and mental health of patients [4]. A total of 25 to 50% of women with infertility are clinically treated for endometriosis, and 30 to 50% of women with endometriosis suffer from infertility [5, 6]. However, the exact link between endometriosis and infertility is unknown, and many factors may be involved in this link. For example, mechanical disruption by pelvic adhesions in women with advanced endometriosis affects oocyte release and transport, decreases sperm motility, and impairs zygote implantation, which leads to reduced fertility [7]. However, the causes of infertility in women with mild endometriosis remain unclear and are subject to numerous speculations, mainly relating to endocrine abnormalities, immune disorders, oxidative stress, and aberrant gene expression [8, 9].

Ferroptosis is a novel form of regulated cell death that is distinct from accidental cell death; it can be mediated by different molecular signalling pathways [10, 11]. Specifically, ferroptosis is defined as an iron-dependent regulated form of necrosis that is caused by massive lipid peroxidation-mediated membrane damage, and this regulated necrosis plays a crucial role in the development and disease of various organisms [12, 13]. Although many open questions remain in ferroptosis research, numerous reports have stated that ferroptosis is closely related to many diseases, such as cancer, ischaemic organ injury, and degenerative diseases [14]. In several recent reports, ferroptosis was detected in ectopic endometrial tissue in endometriosis characterized by periodic haemorrhage [15] and in the early embryo in iron-overloaded peritoneal fluid [16]. However, the specific role and mechanism of ferroptosis in endometriosis, as well as in endometriotic infertility, remain unclear. In this article, we explored the possible mechanisms of the formation of an iron-overloaded environment in endometriotic ectopic lesions, peritoneal fluid and follicular fluid. In addition, we summarized the main pathways and regulatory mechanisms of ferroptosis and discussed its involvement in endometriosis and endometriosis-related infertility to provide new insights into the discovery of novel therapeutic targets.

We propose the notion that a threshold exists for the occurrence of ferroptosis in ectopic endometrial tissue in endometriosis. Once beyond the threshold, iron overload and oxidative damage can lead to ferroptotic cell death. Multiple oxidative and antioxidant systems can be activated simultaneously and operate in parallel to adjust this threshold, which is implicated in the metabolic reprogramming of the affected cells [17]. On the one hand, ectopic endometrial tissues in patients with endometriosis present resistance to ferroptosis, probably because of the shared antioxidant system in macrophages and ectopic lesion cells in the peritoneal fluid. On the other hand, ectopic endometrial tissue is partially subjected to ferroptosis, which seems beneficial. However, this process is followed by the activation of a series of downstream signalling pathways and the release of cytokines that promote cell proliferation. Thus, ectopic endometrial tissue might shift the threshold at which ferroptosis occurs by metabolic reprogramming towards a proliferative advantage for itself, something that seems to be similar to that of cancer cells. However, the specific metabolic checkpoints of the altered thresholds need further exploration, which is a future research direction.

## Ferroptosis

**Fig. 1:**
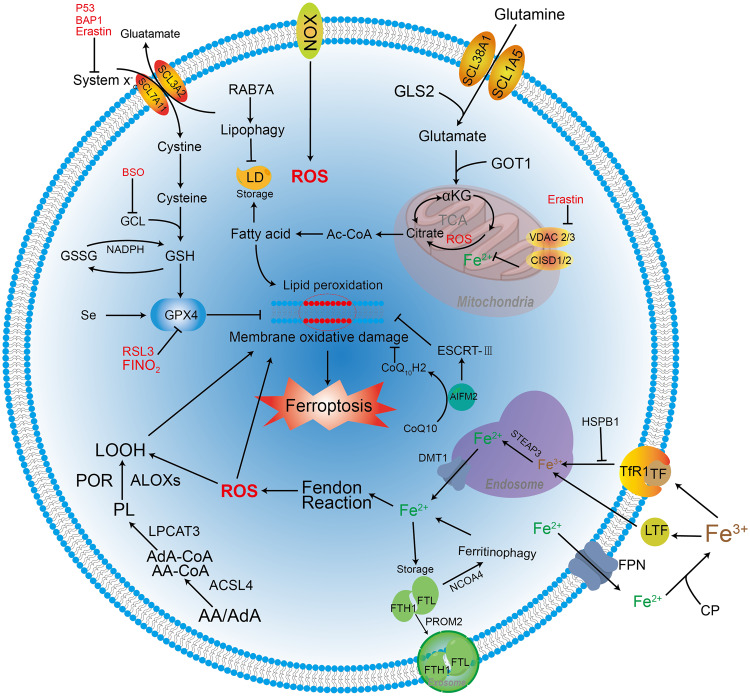
Molecular machinery and regulation of ferroptosis. The molecular machinery of ferroptosis involves cellular antioxidant and oxidative systems, and the regulation of ferroptosis includes iron metabolism and lipid peroxidation.

Dixon et al. first defined ferroptosis as a distinct iron-dependent form of non-apoptotic cell death in 2012 [11]. Ferroptosis is morphologically, biochemically, and genetically distinct from necrosis, apoptosis, and autophagy, and these differing features include abnormal mitochondrial membrane density, iron accumulation, lipid peroxidation, overexpression of ferroptosis biomarkers, and death of leucocyte subsets and the corresponding loss of immune function [11, 18–21]. Of note, ferroptosis that occurs within a cell can spread in a population of cells in a peroxidized lipid and iron-dependent manner [22]. Overall, the core molecular machinery of ferroptosis is regulated by various cellular signalling pathways and genes but is primarily mediated through two main pathways, namely, extrinsic or transporter-dependent pathways (e.g., reduced cysteine or glutamine uptake and increased iron uptake) and intrinsic or enzyme-regulated pathways (e.g., inhibition of glutathione (GSH) peroxidase 4 (GPX4) antioxidant system) (Fig. 1).

### GPX4 pathway

GPX4 is a key factor in the antioxidant system that is regulated by multiple molecular mechanisms. In most cells, cysteine is obtained through the system xc- antiporter, which exchanges extracellular cystine with intracellular glutamate [23]. However, the deletion of the cystine/glutamate antiporter SLC7A11 in mice is well tolerated under unstressed environments [24], indicating that average cells have a low intake requirement for cystine. The activity of exogenously ingested cystine and glutamate–cysteine ligase (GCL) can regulate the synthesis of GSH, which is a major endogenous antioxidant [11, 25]. When GSH exerts an antioxidant effect, GSH can act as an electron donor and oxidize itself to the glutathione disulfide (GSSG) form. The GSH/GSSG ratio usually indicates the level of cellular oxidative stress, which accelerates the conversion of GSH to GSSG and decreases the GSH/GSSG ratio [26].

Based on its unique functions, GPX4 is considered a powerful antagonist of ferroptosis and plays a crucial role in regulating ferroptosis. As a key antioxidant system enzyme, GPX4 can catalyse the reduction in lipid peroxides in complex cellular membrane environments [27]. It can detoxify cellular lipid peroxidation by using the cofactor (GSH) by converting complex toxic lipid hydroperoxides, such as phospholipid hydroperoxides and cholesterol hydroperoxides, into their corresponding nontoxic lipid alcohols. The outcome is that GPX4 reduces the accumulation of ROS and acts against complex lipid peroxidation to reduce cell death [27, 28]. In addition, GPX4 is a kind of selenoprotein. Therefore, GPX4 synthesis is regulated by selenium (Se). Se protects neurons by activating the transcription factors TFAP2c and Sp1 coordinately. This, in turn, upregulates GPX4 and other genes to prevent fatal seizures [29]. Moreover, supplementation with Se could enhance the expression of GPX4 in follicular helper T cells and increase the number of helper T cells to improve the antibody responsiveness of immunized mice after vaccination [30], indicating that the regulation of Se on GPX4 plays an essential role in normal mammalian embryos. Selenocysteine (Sec) is the substitution of Se for sulfur from cysteine, which can enhance the resistance of GPX4 to irreversible peroxidation and prevent hydroperoxide-induced ferroptosis [13, 31].

Throughout the antioxidant system, the regulation of multiple inhibitors and ferroptosis inducers has been implicated. Inhibition of the GCL by buthionine sulfoximine (BSO) induces ferroptosis alone or enhances the sensitivity of cells to ferroptosis induced by other agents [32]. The activity of SLC7A11 is regulated by several factors, such as the transcription factor activating transcription factor 4 (ATF4) and/or nuclear factor erythroid 2-related 2 (NRF2) [33, 34], the epigenetic regulation-associated enzyme BAP1 [35], the tumour suppressor protein p53 [36], the autophagy mechanism component BECN1 [37], and the ferroptosis inducer erastin [38]. RSL3 can directly inhibit GPX4 activity but not its precursor GSH [27]. However, FINO2 indirectly inhibits GPX4 enzymatic function and directly induces ferrous (Fe^2+^) production [39].

### Mitochondria-related pathways

Reactive oxygen species (ROS) are a byproduct of aerobic metabolism that are mainly derived from mitochondrial metabolism and nicotinamide adenine dinucleotide phosphate (NADPH) oxidase (NOX) on the cell membrane, and excessive ROS or the inappropriate location of ROS can damage cells [40]. ROS production in the mitochondria has been shown to be the signalling pathway that regulates the immune response and autophagy but is also important for the induction of ferroptosis [41, 42]. Mitochondria can promote the progression of cysteine-deprivation-induced ferroptosis but not inhibit GPX4-induced ferroptosis [18]. The metabolic network of ROS production can participate in ferroptosis. The transporter SLC38A1 and the amino acid transporter SLC1A5-mediated glutamine uptake and subsequent glutaminase 2 (GLS2)-mediated glutamate production are required for cysteine-deprivation-induced ferroptosis [43, 44]. Glutamate generates α-ketoglutarate (αKG) in mitochondria through transamination by the transaminase GOT1 [44]. αKG can generate acetyl-CoA, a metabolic precursor for lipid synthesis in the cytoplasm, and stimulate dihydrolipoamide dehydrogenase to produce mitochondrial ROS and increase local iron levels to promote ferroptosis [45]. In addition, the tricarboxylic acid cycle or electron transfer chain in the mitochondria can promote lipid ROS accumulation and is involved in cysteine-deprivation-induced ferroptosis [18]. However, ferristatin-1 can specifically prevent ferroptosis induced by erastin, but mitochondria are not involved in the function of ferristatin-1, suggesting that mitochondria may not be necessary for ferroptosis [46].

Notably, the voltage-dependent anion channel (VDAC) in the mitochondrial outer membrane, also known as the mitochondrial pore, acts as a gatekeeper for the entry and exit of mitochondrial metabolites and is a convergence point for its binding to various ligands and proteins to mediate various cell survival and cell death signals [47]. Erastin can directly bind to VDAC 2 and alter mitochondrial membrane permeability, thereby inducing nonapoptotic cell death [48]. Iron–sulfur cluster protein CDGSH iron sulfur domain (CISD) 1, a mitochondrial outer membrane protein, regulates VDAC in a redox-dependent manner in cells and closes mitochondrial pores to prevent iron accumulation in the mitochondria [49]. Nedd4 can be induced upon erastin treatment in melanoma cells, and Nedd4 leads to VDAC 2/3 ubiquitination and mitochondrial pore degradation [50]. These findings all illustrate that VDAC plays an important role in ferroptosis.

### Regulation of ferroptosis

#### Iron-related pathways

Iron is an indispensable metal for the body and is essential for maintaining biological homoeostasis. Iron oxidation has two states, Fe^2+^ and ferric (Fe^3+^), which are mainly present intracellularly and extracellularly, respectively. The interconversion between Fe^2+^ and Fe^3+^ can either donate or accept electrons. This is a process that provides the premise for redox reactions and may affect the sensitivity of cells to ferroptosis. Interestingly, only iron, and not other metals, such as zinc, that also cause ROS generation via the Fenton reaction [51], can induce ferroptosis. Fe^3+^ can bind to transferrin (TF) in serum and is subsequently taken up by the TF receptor 1 (TfR1), which is encoded by TFRC on the cell membrane [52]. Similarly, lactotransferrin (LTF) on cancer cell membranes promotes ferroptosis by increasing intracellular iron levels [53]. Protein kinase C-mediated heat shock protein beta-1 (HSPB1) phosphorylation can stabilize the actin cytoskeleton, thereby inhibiting TfR1-mediated iron uptake and reducing lipid ROS production to limit ferroptotic cell death [54]. Subsequently, Fe^3+^ taken up into the cell is reduced to Fe^2+^ by STEAP3 metalloreductase in the endosome and is then released into the labile iron pool of the cytoplasm by divalent metal transporter 1 (DMT1) [55]. Fe^2+^ participates in various cellular metabolic and biochemical reactions and maintains cellular homoeostasis. The NFS1 cysteine desulfurase can promote iron–sulfur cluster biosynthesis. This results in increased Fe^2+^ availability to inhibit erastin-induced ferroptosis in lung tumour cells and attenuates dihydroartemisinin-induced ferroptosis in leukaemia cells [55, 56]. The CISD1 protein and CISD2 protein, which are present in mitochondria and the endoplasmic reticulum (ER), inhibit ferroptosis by reducing iron uptake from mitochondria and ROS production, respectively [57, 58]. The iron storage protein ferritin consists of ferritin light chain (FTL) and ferritin heavy chain 1 (FTH1) and functions to store iron in cells [59]. This protein can create an iron-overloaded environment and lay the foundation for the occurrence of cellular ferroptosis. Interestingly, RSL3-induced ferroptosis could be inhibited by higher expression levels of mitochondrial ferritin under hypoxic conditions [60]. Moreover, the nuclear receptor coactivator 4 (NCOA4)-mediated selective autophagy pathway (ferritinophagy) increases cellular labile iron pool levels to promote the rapid intracellular accumulation of ROS, which is critical for ferroptosis [61].

Ferroportin1 (FPN1), the only identified mammalian nonhaem iron exporter, can transport Fe^2+^ from intracellular to extracellular spaces [62], and Fe^2+^ is subsequently oxidized to Fe^3+^ by the ferroxidase ceruloplasmin (CP) [63]. Erastin can decrease FPN1 expression and induce iron accumulation in ectopic endometrial stromal cells (ESCs) of women with endometriosis to promote ferroptosis [38]. Knockdown of FPN1 can promote ferroptosis in Alzheimer’s disease (AD) and induce AD-like hippocampal atrophy and memory deficits. Furthermore, differentially expressed genes of the ferroptosis-associated RNA-seq dataset are highly enriched in gene sets associated with AD [62]. Moreover, prominin-2, a member of the prominin family of pentaspan membrane glycoproteins, can mediate the release of ferritin into the extracellular space by exosomes in breast epithelial and breast cancer cells, thereby promoting cellular resistance to ferroptosis [64].

#### Lipid metabolism pathways

Lipids are not only important components of cell membranes but also precursors of various molecules that play important biological roles. However, the excessive accumulation of lipids has potentially toxic effects on individual cells, as well as on the whole body. Previous studies suggest that the peroxidation of polyunsaturated fatty acids in phospholipids by lipoxygenases (ALOX) is particularly important for ferroptosis [65, 66]. After lipid peroxidation occurs, the initiated generation of lipid hydroperoxides (LOOH) and subsequent generation of malondialdehyde (MDA) and 4-hydroxynonenal (4HNE) increase during ferroptosis, leading to a sustained oxidative stress response [67, 68]. Arachidonic acid (AA) and adrenic acid (AdA) are the main substrates of lipid peroxidation in ferroptosis [19], and the lipid peroxidation process involves three enzymes, namely, acyl-CoA synthetase long-chain family member 4 (ACSL4), lysophosphatidylcholine acyltransferase 3 (LPCAT3), and ALOX. ACSL4 binds to AA/AdA and catalyses the formation of AA/AdA-CoA; this is followed by the LPCAT3-mediated esterification of AA/AdA-CoA to phospholipids (PL). Finally, ALOX catalyses the generation of LOOH from PL to promote ferroptosis [69]. Cytochrome P450 (CYP450) oxidoreductase can promote lipid peroxidation by accelerating the cycling between Fe^2+^ and Fe^3+^ in the CYP450 haem fraction and is identified as the alternative source of ROS that induces ferroptosis-related lipid peroxidation [70]. Lipid droplets (LDs) generated from the ER can store lipids in cells and supply lipids for cellular metabolism. The LD cargo receptor RAB7A can mediate selective autophagy (lipophagy) to degrade LDs, which increases the production of free fatty acids and promotes lipid peroxidation. Thus, it ultimately leads to ferroptosis [71].

#### Summary

Under normal physiological conditions, iron plays an important role in metabolic processes. However, whenever the transporter is mutated or deleted, it will disrupt the iron balance and lead to excessive accumulation, triggering cellular oxidative damage and death [72, 73]. Similarly, ROS produced in normal physical processes play an important role in the maintenance of cell function, but excessive ROS may cause metabolic disorders, such as lipid peroxidation, and induce ferroptosis [74, 75]. GPX4 can inhibit ferroptosis by virtue of its special restorative function. Its depletion can lead to a decrease in the antioxidant capacity of cells and increase their sensitivity to ferroptosis [76]. In addition, as an NADPH-dependent coenzyme Q (CoQ) oxidoreductase, apoptosis-inducing factor mitochondria-associated 2 (AIFM2) can use NADPH to catalyse the regeneration of CoQ10 and act synergistically with GPX4 and GSH to inhibit phospholipid peroxidation and ferroptosis [77, 78]. GTP cyclohydrolase-1 (GCH1) can catalyse GTP to tetrahydrobiopterin to exert endogenous antioxidant effects and inhibit ferroptosis [79]. Dihydroorotate dehydrogenase (DHODH) is a flavin-dependent mitochondrial enzyme that can work in parallel with GPX4 to resist mitochondrial ferroptosis [80]. The upregulation of the tumour suppressor gene P53 leads to the accumulation of lipid hydroperoxides by inhibiting the expression of SLC7A11 and reducing the level of GSH, eventually triggering ferroptosis [81]. Ferroptosis is regulated by various factors and pathways.

In short, ferroptosis is an iron-dependent lipid peroxidation form of regulated cell death. Iron metabolism and lipid generation, storage, and degradation are all closely associated with ferroptosis. Excessive free iron levels and dysregulated lipid metabolism in cells trigger ferroptosis, but the oxidative and antioxidant systems are also involved in the regulation and maintenance of cellular processes. In addition, ferroptosis is regulated by many other factors. The complex mechanisms involved need to be elucidated further to help us better modulate the degree of ferroptosis caused by drugs or gene regulation for the treatment of diseases.

## Iron-overloaded environment in endometriosis

Endometriosis can be divided into three phenotypes due to the diverse of underlying aetiologies: superficial peritoneal endometriosis, ovarian endometriosis, and deep infiltrating endometriosis [82]. Studies have shown that the levels of iron, ferritin, and haemoglobin are higher in the peritoneal fluid of women with endometriosis than in that of normal women [83]. Moreover, iron aggregates are present in endometriotic lesions of women with endometriosis and model mice [84, 85]. In addition, ovarian endometriomas contain high amounts of free iron, and the surrounding follicles nearby are also iron overloaded, which adversely affects oocyte development and quality [86]. However, the original cause of the iron-overloaded environment in ectopic lesions, peritoneal fluid, and follicular fluid of endometriosis is still unknown and may be related to the excessive degradation of red blood cells and increased influx caused by menstrual reflux and repeated bleeding of local lesions [87].

Retrograde menstruation and ectopic endometrial bleeding lesions can transport menstrual endometrial tissue and red blood cells to the peritoneal cavity. Some of these tissues and cells will be phagocytized, absorbed, and degraded by peritoneal macrophages and stored in the form of haemosiderin. Additionally, ferritin and haemoglobin are released into the peritoneal fluid [83]. The haem released by the hydrolytic digestion of haemoglobin is catabolized by haem oxygenase to generate active iron and forms iron-ferritin deposition. This overwhelms the iron homoeostasis and iron clearance system, finally leading to an iron-overloaded environment in peritoneal fluid and ectopic lesions of endometriosis [88]. In the environment of intraperitoneal iron overload, excess iron is transported by peripheral TF to cells within the ovary. This iron can bind to TfR1 on the surface of cells and trigger endocytosis [89]. In addition, menstrual reflux to the ovary and repeated bleeding in local lesions of the ovary may also lead to an iron-overloaded environment in follicular fluid. Excessive accumulation of intraperitoneal iron can lead to the overproduction of ROS and the enhanced activation of nuclear factor-kappaB (NF-κB). This enhances the migration ability of human endometriotic cells by promoting the expression of matrix metalloproteinases (MMPs), aggravating inflammation, angiogenesis, and cell adhesion to participate in the progression of endometriosis lesions [59]. Moreover, iron overload in follicular fluid can cause granulosa cell death and affect oocyte maturation and quality, ultimately increasing the risk of endometriosis-related infertility [89, 90].

## Ferroptosis and endometriosis

### The crosstalk between ferroptosis and inflammation

Endometriosis is a chronic inflammatory disease that is closely related to inflammation and the immune response. As a regulated form of cell death, ferroptosis can activate different downstream pathways and complex molecular effector mechanisms, leading to cell lysis in different forms and resulting in morphological changes and immune responses [10].

**Fig. 2:**
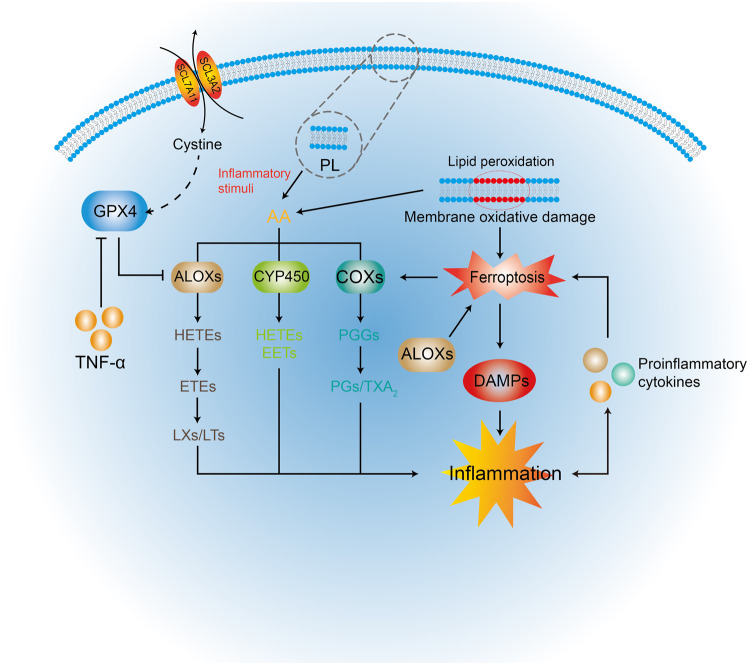
The crosstalk between ferroptosis and inflammation. AA is released from PL by inflammatory stimuli or by intercellular lipid peroxidation. The ALOX, COX, and CYP450 pathways promote further AA metabolization to inflammatory mediators. COX2 expression is increased by ferroptosis. ALOX can promote ferroptosis by catalysing the generation of LOOH. The large array of oxidized lipid mediators released by ferroptosis can contribute to the activity of COX and ALOX. GPX4 inhibits the activity of ALOX and COX directly by decreasing cellular redox states. Ferroptosis initiates inflammatory responses by releasing DAMPs that are immunogenic. Several proinflammatory cytokines play important roles in the crosstalk between ferroptosis and inflammation. For example, TNF can inhibit the activity of GPX4 to promote ferroptosis.

As the main substrate of lipid peroxidation released from PL in the cell membrane, AA is a precursor of proinflammatory mediators that can be metabolized by cyclooxygenases (COX), ALOX, and CYP450 monooxygenases to synthesize biologically active inflammatory mediators, such as prostaglandins (PGs) and leukotrienes [91]. Interestingly, ferroptosis induced by erastin or RSL3 can increase the expression of PTGS2 encoding COX2 [27]. Thus, ferroptosis can promote AA metabolism and inflammatory cytokine secretion via COX2 synthesis. The inactivation of the ferroptosis regulator GPX4, which can upregulate 12/15-ALOX and COX1 expression [92, 93], may accelerate AA metabolism and further promote inflammatory responses. Conversely, the release of inflammatory cytokines promotes the progression of ferroptotic cell death, such as the inhibition of GPX4 expression in tumour necrosis factor-α-treated cells (Fig. 2) [94]. Thus, there is crosstalk between ferroptosis and inflammation.

Similar to nonsilent immune forms of regulated necrosis, ferroptotic cell death can release damage-associated molecular patterns (DAMPs) that promote the development of multiple inflammatory diseases and trigger the innate immune system. These DAMPs can drive tissue inflammation and inflammation crosstalk with ferroptosis. This further promotes an autoamplification loop that exaggerates inflammation and cell death and leads to a more severe degree of cell death and a range of inflammation-related responses [95, 96]. For example, high mobility group box 1 (HMGB1), as a DAMP, is released in an autophagy-dependent manner by ferroptosis inducers and mediates inflammatory responses through the HMGB1-advanced glycation end-product-specific receptor (AGER) pathway, a pathway that activates the NF-κB pathway in innate immunity [94]. This promotes the expression of MMPs and aggravates inflammation, angiogenesis, and cell adhesion in endometriosis.

### Double-edged roles of ferroptosis in endometriosis

Endometriosis is also an oestrogen-dependent gynaecological disease in which excessive oestrogen signalling transduction and altered oestrogen signalling pathways play an important role in its pathogenesis, resulting in oestrogen dominance and progesterone resistance [97, 98]. Oestrogen dependence may be due to the upregulation of the 17β-hydroxysteroid dehydrogenase-1 and aromatase genes, whereas progesterone resistance may result from the failure of progesterone receptor activation and transcription of progesterone target genes [99]. In normal endometrial tissue, oestrogen may inhibit autophagy in the endometrium by inhibiting the hypoxia-inducible factor-1/ROS/AMP-activated protein kinase signalling pathway and further activating mammalian target of rapamycin complex (mTOR) signalling during nonmenstrual periods [100]. However, the level of ROS is no longer suppressed by oestrogen in ectopic endometrial tissue cells. Thus, the level of ROS in ectopic endometrium is notably higher than that in normal eutopic endometrium. Perhaps this is because of the iron-overloaded environment in ectopic tissue cells [101].

The imbalance of iron metabolism plays an important role in the pathogenesis of endometriosis, and studies have confirmed that iron overload exists in the peritoneal fluid of patients with endometriosis [83, 102]. This phenomenon may be related to the increased degradation of red blood cells caused by menstrual reflux [87]. Overloaded iron generates a large amount of ROS by inducing the Fenton reaction, forming an imbalance between antioxidants and leading to oxidative stress reactions such as cellular oxidative damage [83]. Therefore, ectopic endometrial cell proliferation [103], the inflammatory response in the peritoneal cavity [104] and damage to the ovary and its cortex develop [105]. This iron-overloaded and peroxidative environment creates the conditions for the ferroptosis of ectopic endometrial tissue to occur in endometriosis. Li et al. found that a ferroptosis inducer could induce ferroptosis in ectopic endometrial stromal cells through ferroportin-mediated iron accumulation and then alleviate the ectopic lesions of endometriosis. However, the inducer had little effect in normal endometrial stromal cells [38]. The difference might be closely related to the special microenvironment of iron overload in ectopic endometrial stromal cells.

However, the role of ferroptosis in endometriosis appears to be bidirectional. On the one hand, ferroptosis inducers can promote ferroptosis in ectopic endometrial stromal cells, and thus, these inducers may become potential drugs for the treatment of endometriosis. On the other hand, ferroptotic endometrial stromal cells can release inflammatory cytokines and activate downstream regulatory pathways to promote proliferation and angiogenesis in surrounding tissues. Iron overload in ectopic endometrial stromal tissues from patients with ovarian endometriosis-induced ferroptosis, which promoted fibrosis and tissue adhesions, and the process was associated with endometrial stromal cell subpopulations [106]. In a recent study, Li et al. found that ferroptosis in ectopic endometrial stromal cells in patients with ovarian endometriosis could activate the p38 mitogen-activated protein kinase (p38 MAPK)/signal transducer and activator of transcription (STAT) 6 signalling pathway, thereby promoting local upregulation of vascular endothelial growth factor A (VEGFA) and interleukin-8 (IL-8) in ectopic lesions [15]. VEGFA and IL-8 could promote cell proliferation, adhesion, and angiogenesis of ectopic endometrial tissue, thereby promoting the development of endometriosis [107, 108]. In addition, ferroptosis, as a form of inflammatory cell death, is associated with the release of DAMPs, which can trigger the innate immune system and activate the NF-κB pathway through AGER [95, 109]. The excess of Fe^2+^ in ectopic ESCs generates ROS via the Fenton reaction, which contributes to the migration abilities of MMPs via the ROS-NF-κB pathway in ectopic endometrial cells [59]. The overproduction of ROS alters gene expression by regulating the redox-sensitive transcription factor NF-κB. NF-κB-mediated gene transcription in endometriotic cells promotes inflammation invasion, angiogenesis, and cell proliferation and inhibits the apoptosis of endometriotic cells. These effects favour the development and maintenance of endometriosis [110, 111].

**Fig. 3:**
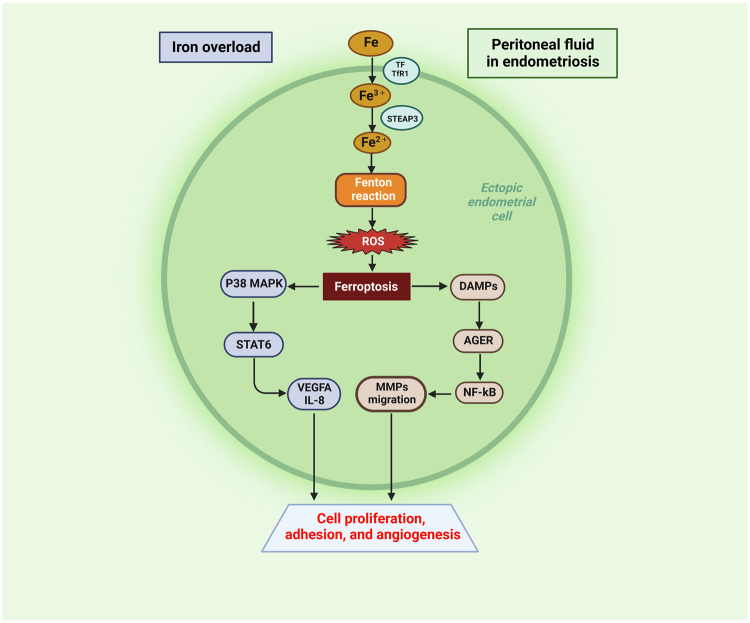
Ectopic endometrial cells in iron-overloaded peritoneal fluid in endometriosis. Iron-overloaded peritoneal fluid results in excess Fe^2+^ in ectopic endometrial cells. Excess Fe^2+^ generates ROS via the Fenton reaction, which contributes to ferroptosis in ectopic endometrial cells. Ectopic endometrial cells promote angiogenesis and cell proliferative adhesion through downstream DAMPs and the P38 MAPK/STAT6 pathways of ferroptosis. Created with BioRender.com.

Iron overload and ferroptosis do occur in endometriotic lesions, and the use of ferroptosis inducers may be a potential treatment for endometriosis. However, a series of downstream inflammatory pathways activated after ferroptosis cannot be ignored, and these pathways further promote angiogenesis and focal fibrosis (Fig. 3). Therefore, in the process of developing ferroptosis-related drugs with the potential to target endometriosis, a series of downstream reactions caused by ferroptosis in ectopic endometrial tissue should be considered, and these issues need to be further resolved in the future.

## Macrophage ferroptosis and endometriosis

Ferroptosis releases DAMPs and lipid oxidation products, which affect nonleukocytes and cause inflammatory cell death. However, it also mediates immune cell death that leads to losses of immune function, such as macrophage function. Macrophages phagocytose aged erythrocytes and process iron from erythrocytes to participate in iron metabolism. Excessive erythrophagocytosis leads to iron overload in macrophages and induces iron-dependent ferroptosis. Iron overload in bone marrow-derived macrophages can upregulate SLC7A11 expression via the ROS-NRF2-antioxidant response element (ARE) axis to reduce cellular sensitivity to ferroptosis [112]. In contrast, mice with GPX4-deficient bone marrow macrophages are susceptible to cell death caused by polymicrobial infection [113]. Furthermore, the release of DAMPs mediated by ferroptosis can affect macrophage polarization, and polarization imbalance can lead to various diseases or inflammatory conditions. For example, Kras^G12D^ released from autophagy-dependent ferroptotic cancer cell death can limit the antitumour effects of macrophages by activating STAT3-mediated AGER-dependent M2 macrophage polarization [114]. Similarly, ferroptosis-mediated cell death that results in the release of 8-hydroxylamine (8-OHG) activates the STING1-dependent inflammatory pathway in surrounding macrophages and promotes M2 polarization [115]. Thus, ferroptosis directly impairs macrophages through the release of DAMPs.

Macrophages play an indispensable role in the chronic inflammatory disease mechanism of endometriosis. Previous studies have shown that macrophages allow the growth of ectopic endometrial tissue, promote angiogenesis, and recruit nerve fibres to contribute to chronic pain [101]. In the human peritoneal cavity, macrophages consist of 50% leucocytes [116]. Unlike other cells that acquire Fe^2+^ through TfRC and DMT1, the major source of iron for macrophages is through the disposal of haem-derived iron. Although macrophages have a remarkable ability to tolerate iron overload [117], the antioxidant capacity of macrophages is insufficient to cope with iron overload in this setting. This ultimately leads to the outcome of ferroptosis due to excessive phagocytosis of erythrocytes and ferritinophagy [118]. Activated M1 macrophages are more sensitive to ferroptosis than M2 macrophages, and this difference is associated with inducible nitric oxide synthase in M1 macrophages [119]. Therefore, iron overload in the peritoneal fluid may promote M2 macrophage polarization, inhibit the M1 macrophage phenotype and induce a subset of macrophage ferroptosis. Recent findings suggest that the M2 macrophage phenotypes with tissue repair effects predominate in the peritoneal fluid in women with endometriosis [120]. Therefore, the peritoneal environment possibly promotes ectopic endometrial tissue proliferation and growth by influencing macrophage M2 polarization via iron overload, which releases anti-inflammatory cytokines, growth factors, and other reparative components [121]. In summary, the intrinsic association between macrophages and endometriosis is much less well-studied than that for other diseases, such as cancer. The mechanisms by which macrophages resist ferroptosis help provide us with new insights into the mechanisms of ferroptosis in the endometriosis model.

## Ferroptosis and endometriosis-related infertility

The iron-overloaded environment induced by retrograde menstruation is suspected to be an important factor in inducing the continued proliferation of ectopic endometrial tissue. In addition, ferroptosis promoted by an iron-overloaded environment appears to be detrimental to oocytes or embryos and is also closely related to endometriosis-related infertility. Peritoneal fluid and follicular fluid are the external microenvironments for oocyte maturation and blastocyst formation, and these abnormal microenvironments affected by iron overload may lead to impaired reproductive function.In recent years, studies on the role and mechanism of iron overload and ferroptosis in endometriosis-related infertility have been reported successively (Table 1).

**Table 1:** Studies on the association of iron overload and ferroptosis with endometriosis-related infertility.

Author, date (Ref.)	Model	Research content	Main results	Final outcomes
Chen et al., 2021 [104]	In vivo: C57BL/6J female mice In vitro: mouse two-cell stage embryos	Iron overload in endometriosis peritoneal fluid	Disrupted mitochondrial function, decreased ATP levels, increased ROS levels, hyperpolarized MMP, triggered apoptosis and ferroptosis	Compromised preimplantation mouse embryo development
Li et al., 2021 [16]	In vivo: C57BL/6J female mice In vitro: mouse two-cell stage embryos	Iron overload in endometriosis peritoneal fluid	Disrupted blastocyst formation, decreased GPX4 expression, disrupted mitochondrial function, decreased ATP levels, increased ROS levels and hyperpolarized MMP, upregulated HMOX1	Embryotoxicity and early embryo ferroptosis
Ni et al., 2022 [110]	In vivo: Kunming female mice In vitro: mouse granulosa cells and human granulosa cells	Iron overload in endometriosis follicular fluid	Decreased GPX4 and GSH expression, increased NCOA4 expression, NCOA4-mediated ferritinophagy, released exosomes of granulosa cell containing abnormal miRNAs	Ferroptosis of granulosa cells and oocyte dysmaturity
Li et al., 2020 [109]	In vitro: mouse oocytes	Transferrin insufficiency and iron overload in endometriosis follicular fluid	Reduced concentration of transferrin with three analogues, increased concentration of ferricion, decreased maturation in vitro rate of mouse oocytes	Oocyte dysmaturity
Hu et al., 2021 [111]	In vitro: porcine oocytes	Iron overload-induced ferroptosis in porcine oocytes	Increased intracellular ROS generation, decreased intracellular free thiol levels, induced mitochondrial dysfunction, triggered autophagy, decreased embryonic developmental potential	Impaired oocyte meiosis, decreased oocyte quality and embryonic developmental competence
Ding et al., 2022 [112]	In vivo: C57BL/6J female mice	Iron overload in endometriosis ovarian function	Increased MDA levels, decreased GPX4 and GSH expression, decreased growing follicles numbers	Cellular ferroptosis, compromised ovarian function

Iron overload in peritoneal fluid can affect embryonic development by leading to embryo toxicity and ferroptosis. Chen et al. showed that the pelvic iron-overloaded environment in patients with endometriosis impaired early embryonic development and caused embryo toxicity by triggering GPX4 downregulation-dependent ferroptosis in preimplantation mouse embryos. This leads to endometriosis-related infertility and adverse pregnancy outcomes [122]. During this process, excess iron could induce the excessive accumulation of ROS, which leads to oxidative stress and damages mitochondrial function in preimplantation mouse embryos. This triggers ATP generation impairment and decreases mitochondrial membrane potential (MMP) levels. Moreover, the expression of GPX4 in embryos was significantly decreased [122]. GPX4 is essential for embryonic development. GPX4 deficiency results in abnormal embryonic development compared to the deficiencies of all other GPX family members and ultimately produces lethal phenotypes in mice [123]. In addition to disrupting mitochondrial function, the iron-overload environment in the peritoneal fluid of endometriosis could also reduce the expression of GPX4 and induce lipid peroxidation. Thus, blastocyst formation is disrupted, and embryo toxicity and ferroptosis occur. The ferroptosis inhibitor Fer-1 could improve these adverse conditions [16]. In addition, haem oxygenase 1 (HMOX1) is upregulated in embryonic ferroptosis, and inhibition of HMOX1 can maintain normal mitochondrial function, thereby preventing ferroptosis from occurring [16]. Thus, HMOX1 may play an important role in mediating embryo ferroptosis. Its overexpression can play a pro-oxidative role and induce ferroptosis by increasing Fe accumulation and lipid peroxidation [124, 125].

The total iron levels and ferritin and TfR1 expression levels in endometrioma-proximal follicles are higher than those in endometrioma-distal follicles and healthy ovarian follicles. Moreover, the oocyte retrieval rates in endometrioma-proximal and -distal follicles are lower than those in healthy ovarian follicles [126]; this illustrates that excessive iron intake by follicles leads to cytotoxic accumulation that affects normal oocyte development.

In recent research, Li et al. studied specific proteins at different concentrations in the follicular fluid of patients with advanced endometriosis and found that the transferrin concentration of the three analogues of cDNA FLJ53691, cDNA FLJ54111, and TRF variant Fragment in the follicular fluid decreased. The iron ion concentration of these analogues increased. The environment of transferrin deficiency and iron overload could increase the level of ROS and lead to oxidative stress. Thus, the in vitro maturation rate of mouse oocytes could significantly decrease, which might be one of the causes of endometriosis-related infertility [89]. Ni et al. found that iron-overloaded follicular fluid could trigger ferroptosis in granulosa cells and immaturity of oocytes, thereby increasing the risk of endometriosis-related infertility [90]. The iron-overloaded environment of follicular fluid could not only inhibit the expression of GPX4 and its upstream regulatory target GSH but also cause the high expression of NCOA4 in granulosa cells. This would lead to NCOA4-dependent ferritinophagy, which increases lipid peroxidation in granulosa cells and promotes ferroptosis. Moreover, granulosa cells undergoing ferroptosis cannot exert nutritional and paracrine functions on oocytes and can release granulosa cell exosomes containing abnormal miRNAs. Therefore, oocyte maturation is inhibited, and endometriosis-related infertility can develop. The iron chelators deferoxamine mesylate and VITE could change these circumstances by increasing GPX4 expression and decreasing iron overload [90].

Furthermore, after in vitro ferroptosis inducer ferric ammonium citrate (FAC) intervention, mammalian oocytes experienced increases in ROS and autophagy-related protein LC3 and mitochondrial dysfunction. Additionally, there was significant accumulation of Fe^2+^ in the cytoplasm and decreases in the polar body (PB) expulsion rate and blastocyst formation rate. Thus, exogenous ferroptosis inducer-induced ferroptosis inhibits oocyte meiosis by increasing oxidative stress, inducing mitochondrial dysfunction, triggering autophagy splitting process and affecting oocyte quality [127]. Conversely, the inhibition of ferroptosis might not only inhibit the progression of endometriosis, but also improve the adverse effects of iron overload on ovarian function, thereby improving fertility and becoming a therapeutic approach for endometriosis-related infertility [128].

**Fig. 4:**
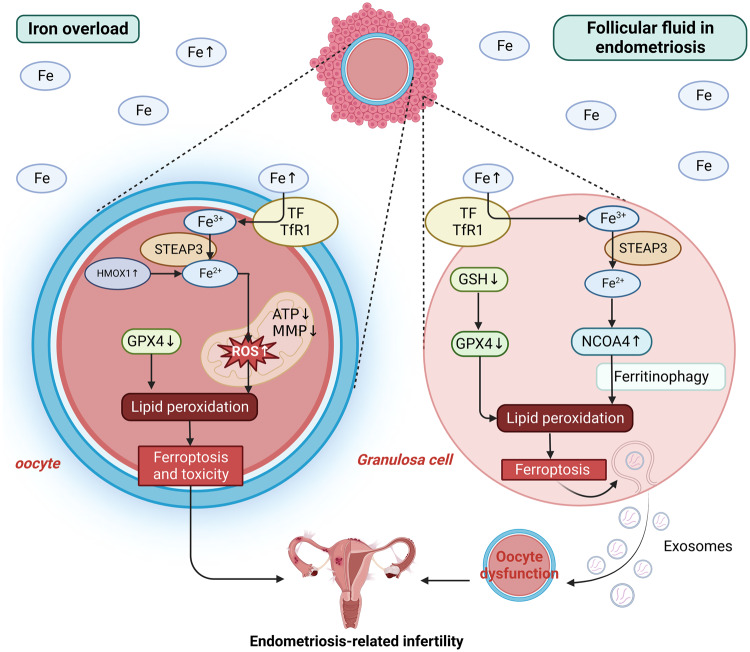
Oocyte and granulosa cells in iron-overloaded follicular fluid in endometriosis. Iron-overloaded follicular fluid in endometriosis plays an important role in the progression of endometriosis-related infertility. Iron overload in peritoneal fluid affects the mitochondrial function of oocytes and decreases GPX4 expression, thereby inducing ferroptosis and toxicity by promoting lipid peroxidation. Moreover, iron overload in follicular fluid not only decreases GPX4 and GSH expression, but also increases NCOA4 expression and mediates ferritinophagy. Thus, granulosa cell ferroptosis is induced by promoting lipid peroxidation. Granulosa cells undergoing ferroptosis cause oocyte dysmaturity by releasing exosomes containing abnormal miRNAs. These situations can contribute to endometriosis-related infertility. Created with BioRender.com.

In summary, these findings suggest that iron overload and its induced ferroptosis in peritoneal fluid and follicular fluid in patients with endometriosis play an important role in the progression of endometriosis-related infertility (Fig. 4). Therefore, mitigating the impact of iron stress on the local microenvironment, such as the use of antioxidant agents or iron chelators, is expected to be an effective approach for the prevention and treatment of endometriosis-related infertility.

## Conclusion

In recent years, researchers have gradually appreciated and revealed the potential role of ferroptosis in endometriosis. These findings highlight the ability of ectopic endometrial tissue to resist iron overload-induced ferroptosis and promote ectopic lesion growth by mediating local cellular ferroptosis in peritoneal fluid in patients with endometriosis. However, oocytes from patients with endometriosis-related infertility are threatened by iron overload, and the development and maturation of oocytes are affected and prone to trigger cellular ferroptosis. This is possibly due to the immature antioxidant system and membrane repair mechanisms of the oocyte. Furthermore, although iron accumulation and lipid peroxidation are unique intermediate events in the onset of ferroptosis, they are not the ultimate executors. Lipid peroxidation can also occur in other cell death types, which depend on different ultimate effectors. Key regulators of ferroptosis can also regulate other types of cell death. For example, GPX4, a key factor in the antioxidant system, also inhibits apoptosis and necroptosis to protect cells from various insults [129, 130]. Therefore, unique markers of ferroptosis in ectopic endometrial tissue require further identification. Currently, the detailed regulatory mechanisms of ferroptosis in endometriosis have not been fully elucidated. In conclusion, ferroptosis and its role in endometriosis, as well as endometriosis-related infertility, require systematic and in-depth studies.
